# Sesotho Women’s Preferences for Male Partner Involvement During Antenatal Care and Delivery

**DOI:** 10.3390/ijerph22121867

**Published:** 2025-12-15

**Authors:** Michelle Engelbrecht, Ngwi Mulu, Gladys Kigozi-Male

**Affiliations:** Centre for Health Systems Research & Development, University of the Free State, Bloemfontein 9300, South Africa; ngwi.mulu@gmail.com (N.M.); kigozign@ufs.ac.za (G.K.-M.)

**Keywords:** male partner involvement, maternal health, antenatal care, delivery

## Abstract

**Highlights:**

**Public health relevance**

- Women’s perspectives on male partner involvement (MPI) are essential for informing maternal health interventions that align with community norms and expectations.
- Exploring these preferences among Sesotho-speaking women provides insight into how cultural norms, lived experiences, and health service conditions shape attitudes towards MPI during antenatal care (ANC) and delivery.

**Public health significance**

- Most women supported men’s attendance at ANC visits and delivery, emphasising emotional reassurance, practical assistance and improved partner awareness of pregnancy related risks.
- Preferences for MPI were influenced by previous partner involvement, maternal age, and concerns regarding privacy, cultural expectations, and potential distress or judgement during pregnancy.

**Public health implications**

- Interventions to strengthen MPI should be context specific and responsive to women’s needs, promoting supportive forms of involveinstitutional barriers and acknowledging cultural considerations may improve the acceptability and implementation of MPI in maternal health care.

**Abstract:**

Male partner involvement (MPI) is recognised as an important strategy for improving maternal health, yet little is known about women’s preferences for how men should participate in antenatal care (ANC) and childbirth. This study explored Sesotho-speaking women’s preferences for MPI and identified the forms of involvement that they considered to be appropriate and beneficial using a concurrent mixed-methods design. A survey was conducted with 513 women who had children under six years, and eight focus group discussions were held with 64 women of reproductive age. Quantitative data were analysed using descriptive statistics and binomial logistic linear regression, while qualitative data were analysed using inductive thematic analysis. Survey findings revealed that 95.7% of women supported male attendance at ANC visits, while 78% favoured male presence during delivery. Support for MPI during delivery was associated with prior male attendance at ANC and previous birth, as well as older maternal age. Qualitative findings highlighted that women valued emotional and practical support and appreciated men’s improved knowledge about pregnancy and childbirth. However, some women expressed concerns about privacy, cultural expectations, male anxiety, and potential verbal abuse. The combined findings reveal the complexity of women’s perspectives on MPI, underscoring the importance of context-sensitive interventions that prioritise women’s voices while promoting constructive male engagement in maternal health.

## 1. Introduction

Male partner involvement (MPI) in maternal health is widely recognised as a strategy for improving maternal and newborn health outcomes [1,2,3,4,5]. Evidence shows that MPI can reduce the risk of maternal depression, enhance women’s use of antenatal care (ANC) [3,4], increase skilled birth attendance [2,4], improve birth preparedness [4], and encourage joint decision-making [5]. These benefits are realised when MPI complements rather than undermines women’s autonomy and decision-making [1].

While there is extensive research on men’s perspectives of the barriers to MPI in maternal health, relatively few studies have examined women’s preferences for how and when male partners should be involved [6,7,8]. Women’s views are critical, because the acceptability, safety, and effectiveness of MPI interventions depend on aligning male participation with women’s needs. Existing research from African settings highlights that women’s preferences are not uniform. Some women welcome partner attendance at ANC visits [9,10,11,12,13,14] and during delivery [6,11,12,13,15], while others prefer male absence [6,9,10,16], due to concerns including fears of violence [10], cultural beliefs, upholding patriarchal gender roles [6,14], or the desire to protect women’s social spaces within healthcare facilities [6].

Focusing on studies conducted in the African region provides the most relevant comparative foundation for interpreting women’s preferences for MPI in the present study. In many African settings, maternal health and childcare are traditionally considered to be the woman’s responsibility [6,14,16,17], yet sub-Saharan African women’s participation in decision-making about their own health is among the lowest globally [18], although there is variation across countries [19]. Health services in the region often engage men primarily when their participation benefits women’s health outcomes [20]. Despite this, health services are not necessarily equipped for MPI due to long waiting times at healthcare facilities [17,21,22], poor attitudes of healthcare workers [17,22,23], and poor infrastructure that does not allow men to be accommodated in consultation or delivery rooms [16,17,24].

Studying the preferences of Sesotho-speaking women for MPI in maternal health is particularly relevant in the South African context, where cultural diversity [25] and traditional practices continue to shape maternal health behaviours and contribute to morbidity and mortality [26]. Maternal and child healthcare services are provided free of charge in South Africa for those without medical aid/private healthcare insurance (i.e., 84% of the population) [27,28]. However, most public healthcare facilities in the country do not invite men to attend antenatal care visits or to be present at the birth, regardless of the women’s wishes [9]. Prior research among Sesotho-speaking women identified that pregnancy was perceived as a natural transition, with ANC sought primarily during the later stage of pregnancy. It was also described as a family event requiring preparation for necessary customary practices and for the baby’s arrival. Men play a supportive role through traditional duties such as fetching wood, reinforcing the communal nature of this period [29]. At the same time, pregnancy disclosure is treated with caution, as women often keep the pregnancy secret to protect the unborn child from witchcraft, a concern deeply rooted in many African societies [30]. How these cultural expectations align, and sometimes conflict, with contemporary MPI interventions requires further understanding.

Given this context, the aim of this study was to explore Sesotho-speaking women’s preferences for male partner involvement during ANC and delivery, and to identify the forms of involvement that they considered to be appropriate and beneficial. By foregrounding women’s voices, this research contributes to a more nuanced and contextually grounded understanding of MPI in maternal health.

## 2. Materials and Methods

### 2.1. Research Design and Setting

This study employed a mixed method, concurrent design [31], consisting of a cross-sectional survey and focus group discussions (FGDs) conducted in parallel. This design enabled the researchers to examine the research problem from multiple perspectives and methods, and to triangulate and validate the data, thereby enriching the findings. Data were collected between August and November 2022 in the Mangaung Metropolitan Municipality of the Free State Province, South Africa.

### 2.2. Population and Sampling

The target population for the quantitative component of the study was women who had given birth within the past six years and were residing in Mangaung Metropolitan Municipality at the time of data collection. More specifically, the inclusion criteria were as follows: being Sesotho-speaking; being a biological parent to at least one child younger than six years of age; and being able to provide informed consent. In 2025, there was an estimated 843,977 women of reproductive age (15–49 years) in the Free State Province, of whom approximately 227,873 resided in Mangaung Metropolitan Municipality, and an estimated 164,069 were Sesotho-speaking [32,33,34]. Using this population size, a 5% margin of error, 95% confidence interval (CI), and a response distribution of 50%, the minimum sample size calculated with a digital sample size calculator [35] was 384 women, with a maximum sample not exceeding 662 participants. To compensate for non-response and to increase statistical power, we targeted 520 participants. A combination of purposive and convenience sampling was used to select women who met the inclusion criteria. A total of 513 women were recruited with the assistance of non-governmental organisations, churches, and women’s forums.

For the qualitative component of the study, the target population was women of reproductive age (18–49 years). A total of 64 women were purposively sampled and recruited with the assistance of non-governmental organisations, churches, and women’s forums, where posters and brochures advertising the project were available. Interested women were requested to contact the fieldwork manager or to meet the fieldwork team at designated venues and times.

### 2.3. Research Instruments and Data Collection

The quantitative data was collected by trained female fieldworkers, conversant in Sesotho and English. The fieldworker administered questionnaire was available in both languages and included questions on participant characteristics (i.e., age, marital status, education level, employment status, receipt of social grants and cohabitation), ANC and delivery experiences, and preferences for MPI. Gender norms were measured using the Gender Equitable Men (GEM) scale [36].

Items on the GEM scale are answered on a three-point scale—agree, partially agree, and disagree. The GEM comprises two sub-scales [37]. The first sub-scale focuses on inequitable gender norms and comprises seven items: (1) A woman who has sex before she marries does not deserve respect; (2) It is a woman’s responsibility to avoid getting pregnant; (3) A real man produces a male child; (4) A woman’s role is taking care of her home and family; (5) The husband should decide to buy the major household items; (6) A man should have the final word about decisions in his home; and (7) A woman should obey her husband in all things. The sub-scale had a Cronbach’s alpha coefficient of 0.78.

The second sub-scale focuses on violence, particularly gender-based violence, and includes six items: (1) There are times when a woman deserves to be beaten; (2) A woman should tolerate violence to keep her family together; (3) It is alright for a man to beat his wife/partner if she is unfaithful; (4) A man can hit his wife/partner if she won’t have sex with him; (5) If someone insults a man, he should defend his reputation with force if he has to; and (6) A man using violence against his wife/partner is a private matter that shouldn’t be discussed outside the couple. The sub-scale had a Cronbach’s alpha coefficient of 0.91.

The focus group discussions were conducted in Sesotho by trained female facilitators using a semi-structured focus group guide and focused on women’s preferences for MPI during ANC and delivery, perceived benefits, and challenges. The discussions were held in private community venues, lasted approximately 90 min, were audio recorded with consent, and were transcribed verbatim before being translated into English.

### 2.4. Data Analysis

Quantitative data was analysed in IBM SPSS version 29 [38]. Frequency counts and percentages were calculated to describe categorical variables while means and standard deviations (SD) described continuous variables. Binomial logistic regression was conducted to identify factors associated with women’s belief that male partners should be present in the delivery room. The predictor variables included cohabitation with the father of the youngest child, number of biological children, partner attendance at ANC, partner presence during previous delivery, maternal age, and gender inequitable norms. The results are presented as adjusted odds ratios (AORs) and 95% confidence intervals (CIs). Statistical significance was set at *p* < 0.05.

Qualitative data was thematically analysed using the inductive approach according to Braun and Clarke’s six phases [39]. English transcripts were transferred to NVIVO 14 and repeatedly read to ensure familiarisation with the data. NVIVO supported coding and data management. The second author generated the initial codes and grouped similar codes into categories. The codes and categories were refined, and themes were identified. The trustworthiness of the data was assured by ensuring credibility, transferability, dependability, and confirmability were observed [40] by triangulating the data from different sources (i.e., biological mothers and women of reproductive age), using different research methods (i.e., a survey and focus group discussions) and maintaining an audit trail of the research process.

### 2.5. Integration of Mixed Methods

Quantitative and qualitative results were integrated at the interpretation stage. This triangulation allowed us to identify how women’s preferences (survey) aligned with their narratives and contextual explanations (FGDs), thereby enhancing the explanatory depth of the findings.

### 2.6. Ethical Considerations

Ethical approval was obtained from the Health Sciences Research Ethics Committee of the University of the Free State (ethics approval number: UFS-HSD2022/0304/1_2). All participants provided written informed consent. Privacy and confidentiality were maintained during data collection, and no identifying information were recorded. Participants were provided with the contact details of their closest primary healthcare facility should they have experienced any emotional distress because of participation in the study.

## 3. Results

### 3.1. Participant Characteristics

A total of 513 women with children younger than six years participated in the survey. The mean age was 31.33 years (SD = 6.967). Most women had completed secondary school (84.7%), were unemployed and seeking work (70.7%), and received social grants (92.2%). Two thirds (66.7%) were in a non-marital relationship, and 61% lived with the father of their youngest child (Table 1).

The mean score for the seven-item gender inequity sub-scale was 9.72 (Range, 6–18; SD = 3.328), indicating some variability in participants’ endorsement of inequitable gender norms. The six-item violence sub-scale had a mean of 6.19 (Range = 6–18, SD = 3.09) reflecting low endorsement of statements supporting violence. Item-level responses to the GEM Scale reinforced these findings (Table 2): participants overwhelmingly rejected gender-based violence and most inequitable gender norms, while showing more varied attitudes toward traditional gender roles within the household. Nearly all women disagreed with statements condoning violence against women (more than 97% disagreement across items). In contrast, beliefs related to domestic responsibilities and spousal obedience showed greater variation, with 41.5% agreeing that a woman’s main role is caring for the home and 17.7% agreeing that a woman should obey her husband. These patterns indicate generally low tolerance for explicit inequity and violence, but some endorsement of certain traditional gender expectations.

### 3.2. Antenatal Care

Most women (95.7%) believed it was important for men to accompany their partners to ANC visits, while only slightly more than half (54.6%) indicated that their partner had ever attended ANC visits (Table 3). The reasons why men did not attend ANC visits were lack of time (50.9%); inconvenient clinic hours (42.6%); lack of transport (30.9%); not wanting to have him present at the visit (22.1%); concerns about being stigmatised (20.4%); past negative healthcare experiences (20.2%); negative attitudes of healthcare workers (18.8%); and lack of confidentiality/privacy at healthcare facilities (18.3%).

The most common forms of male support during pregnancy were financial support (84.4%), provision of nutritious food (77.2%), and emotional support (74.3%) during pregnancy. Support with household chores was less frequent (55.6%).

### 3.3. Delivery

Overall, 78% of survey respondents supported male presence in the delivery room, although only 14.8% indicated that their partner was present when they gave birth (Table 4). The main reasons why men were not present were that they had to work (34.7%), with a smaller percentage of women indicating that their partner did not want to be present (15.3%) or that he was scared to be present (14.8%) (Table 3). Women who did not want their partner to be present during the delivery provided the following reasons for this: men are too scared and cannot handle pain (17.9%); men are not supposed to see what happens during the delivery (16.1%); their partner would insult and disrespect them if they saw what happened during the delivery (15.2%); and it would be pointless to have their partner there as he would not be of any help (10.7%).

A binomial logistic regression was performed to determine the effects of cohabitation with the father of one’s youngest child, number of biological children, partner attendance at ANC visit, partner presence during previous delivery, maternal age and gender inequitable norms on women’s belief that it is important for men to be present during the delivery of their baby. The logistic regression model was significant, X2(8) = 32.261, *p* < 0.001). The model explained 11.8% (Nagelkerke R2) of the variance in the tendency to believe that men should be present during the delivery. Sensitivity was 99.7% and specificity was 3.4%. The positive predictive value was 79.25% and the negative predictive value was 75%. Of the six predictive variables, three were statistically significant. Where the father had attended ANC visits, the women were 1.9 times more likely to think that it was important for men to also be present at the delivery compared to instances when the male partner did not attend ANC (OR: 1.914; *p* = 0.013). Where fathers were present at the previous delivery, the women were also 4.5 times more likely to believe that it was important for men to attend the delivery compared to when their partner was not present at the delivery (OR: 4.544, *p* = 0.005). Compared to younger women, older women were more likely to believe that it was important for men to be present at the delivery (OR: 1.067; *p* = 0.013) (Table 5).

### 3.4. Qualitative Findings

Eight FGDs with 64 women provided insight into contextual and cultural factors shaping preferences for MPI. The findings are organised according to women’s preferences for MPI during **antenatal care (ANC)** and **childbirth**, highlighting the types of support desired, reasons for involvement, and factors limiting male participation (see Table 6). Women emphasised the importance of MPI for **emotional support, practical assistance, knowledge and awareness**, and a **protective role** during pregnancy and labour. They also noted that involvement should be **conditional**, respecting privacy, avoiding distractions, and considering community perceptions.

**Theme 1—Emotional support**

The women consistently referred to the importance of emotional support during pregnancy and delivery. They highlighted the opportunity for men to bond with their unborn child, stating, “*I think the father builds relations with the child while still inside you … calling the baby by clan name*” (Group A). This emotional presence extended beyond bonding with the baby to the emotional assurance that women felt from their partners. They described how their partners support during the pregnancy and delivery brought them happiness. One participant expressed that having her partner accompany her to the hospital made her feel deeply valued: “*when it happens… there is nothing that would make me more happier than that*” (Group A). Others shared how meaningful it was to feel loved and supported at home, whether through a foot massage, companionship, or the comfort of knowing someone cared. “*I feel very good and emotionally happy when I am with my husband*,” (Group E).

Male partner presence and support contributed to the women’s mental well-being, reducing stress and easing emotional strain. Many noted that having a supportive partner eased the burden of pregnancy, explaining that he “*will unburden my mind and thoughts*” (Group A), and “*will lift off your burdens at that current situation*” (Group C). Furthermore, supportive partners helped the women to feel less overwhelmed and stressed, which contributed to their overall well-being.

“*If he is always with you, you don’t get to stress right? You won’t even get high blood pressure because that is the cause to all miscarriages or to women not being well*.”

(Group C)

**Theme 2—Practical support**

Women valued male involvement in terms of practical support throughout pregnancy, particularly when it came to transport and attending ANC visits or presence at delivery. They described feeling safer and more supported when their partners accompanied them on long walks to the clinic, *“…it is risky because anything can happen along the way. So if your partner is by your side, you are safe because he can help.*” (Group B). Their presence at clinics also eased the physical strain of long queues, allowing women to rest while their partners “*take over the queue*” (Group B). The women also noted that their partners should assist in cases of emergencies, such as calling the ambulance when it was time to deliver the baby, *“…it’s a long walking distance so at least when he is there even when the pains come, he can be able to call someone or call an ambulance.*” (Group F).

Practical support at home was equally important. Women valued partners who shared household tasks during pregnancy, including cooking washing dishes and carrying heavy items, “*… he must help you maybe you I didn’t wash dishes or cook he will cook and wash dishes*” (Group A). Physical comfort and care were also mentioned, with partners offering massages, helping soak feet, or providing snacks and reassurance. As one participant noted, “*It’s so nice ehhh when a father arrives, he asks you what you are craving for, how have you been and he massages your tummy…*” (Group G)

Financial support formed another key dimension of practical assistance, helping pregnant women maintain healthy diets and purchasing clothing, which reflects how financial stability contributes to a healthier and more comfortable pregnancy experience. One women explained:

“*Pregnant women must eat healthy so that the baby is born healthy as well. He must also buy the mother necessary clothes seasonally*.”

(Group D)

**Theme 3—Knowledge and awareness**

Women appreciated male involvement as a way for men to learn about pregnancy, childbirth, and caring for the baby. It was elaborated that men who attended ANC with their partner had the opportunity to learn about the health risks (e.g., high blood pressure and diabetes) associated with pregnancy, as well as the importance of providing support with household chores such as lifting, cleaning, and cooking to reduce physical strain and risk during pregnancy.

“*He must go with you so that he learns that sometimes as pregnant women you would be healthy before pregnancy then when you are pregnant you become high risk and have high blood pressure, diabetes they all appear*…”

(Group A)

Male partner attendance at ANC visits also provided an opportunity for men to be tested for HIV. The women felt that this was important as knowing one’s HIV status would help in planning for the care of the baby. In addition, women noted they were afraid to tell their partner that they were HIV-positive, and that it was better for him to hear it at the clinic, “… *you get tested and they’d tell you that you are HIV positive, and you will be afraid to tell him so he will hear it too.”* (Group F). By attending together, partners shared responsibility for testing, treatment, and planning for the well-being of both mother and baby.

**Theme 4—Protective role during delivery**

Women emphasised the need for men to be present at the hospital during the delivery to ensure both their own safety (e.g., an emergency caesarean section), and that of the newborn. Some women expressed fears about babies being switched or stolen in hospitals, especially when mothers are unconscious or feeling weak and dizzy after giving birth and noted that “*If your partner is there, he will be able to see what mark a child has to identify him/her …*” (Group B). Women described relying on partners to remember important details from nurses that they themselves might miss “he can explain, what the nurses said when you arrive at home,” (Group H).

Concerns about the treatment received at hospitals, was another important reason for men to be present during the delivery. Women shared experiences of delayed care, lack of attention, or even verbal abuse while in labour, noting that having a partner present could deter such treatment or at least bear witness to it. One woman explained that she wanted the father there so he could “*see the abuse that I am going to receive*” (Group G), while another indicated:

“*In labour we are being abused when you tell the nurse that it’s time, she looks at her watch, she is God I think, she tells you that you will go to labour at 4 at that time your face is purple ma’am, you are in pains and you short of breath.*”

(Group G)

In addition, partners played an important legal role when complications arose. For women who required emergency caesarean sections or other interventions, fathers were needed to sign consent forms and support decision-making. As one participant explained, “you cannot go through that without anyone signing forms for you,” making partner presence essential for both emotional and legal protection during delivery.

**Theme 5—Conditional preferences**

Although many women supported male involvement during ANC and delivery, there were conditions and limitations. Some women preferred that their partners only accompanied them to the clinic gates, explaining that waiting-room conversations among pregnant women often involved sensitive or personal experiences that men did not need to hear. In addition, women who attended ANC visits on their own, might feel hurt that their partner was not present.

*For instance, you find that it is painful for other mothers to see supportive fathers at the clinic because they wish they had that support, but they don’t*.

(Group B)

Similar concerns were raised about male presence during the delivery. Women described discomfort at the idea of being exposed during childbirth, fearing embarrassment, judgement, or even future verbal abuse based on what their partner might witness. Some felt that men could misuse what they saw during labour to insult or shame them during future conflicts, “*he might one day use what he saw to insult me*” (Group D). Others worried that anxious partners would panic or distract health workers, creating additional risk, “*Doctors might even drop the baby because of him*” (Group C). For these women, ANC attendance and childbirth remained a private, women-only space where only nurses and clinical staff should be present, even if they appreciated male involvement in other aspects of pregnancy and parenting.

**Theme 6—Cultural concerns**

Some women described how community perceptions and cultural beliefs discouraged male involvement in pregnancy, childcare, and household responsibilities. Supportive men—those who cooked, washed baby clothes, or accompanied their partners to the clinic—were viewed by some community members with suspicion or ridicule. The women explained that communities sometimes interpreted such behaviour as a sign that a man had been “*fed something*” (Group A) implying he was being controlled through witchcraft or love potions. One woman noted that when a father takes an active role, “*They accuse you of controlling and feeding him a magic potion from witch doctors that controls him to do whatever you want him to do*” (Group B). This was supported by another woman who elaborated, “*He was busy washing spinach and in my mind, I am like yhoo they are going to say I gave him love potion*” (Group E). These cultural narratives not only stigmatised involved fathers but also made some women fear judgment for accepting or encouraging their partner’s support.

## 4. Discussion

MPI in maternal health is significantly shaped by societal norms, personal preferences and prevailing gender relations in households [41]. Given the lack of information on women’s preferences for MPI in maternal health [6,7,8], this paper contributes to addressing the gap by foregrounding “women’s voices” and illustrating how preferences for MPI during ANC and delivery are conditional, context-dependent, and shaped by gendered expectations. While research is limited, earlier studies illustrate that women do not share a uniform view of MPI in maternal health care [6,7,9,14,16]. This is also evident in the current study, the findings reflect a nuanced understanding of when and how women perceive male involvement as helpful, supportive, intrusive, or inappropriate.

Our survey findings showed overwhelming support for male partners to attend ANC visits consistent with earlier South African [9] and wider African [11,12,13,14,42] studies. The qualitative data added depth to these findings by illustrating why women preferred MPI during ANC, specifically mentioning issues related to emotional and practical support, as well as improved knowledge and awareness about pregnancy and childbirth. Women emphasised that emotional support from partners helped reduce stress during pregnancy, which was confirmed by prior South African [9] and Kenyan research [7]. Practical support, particularly financial support (i.e., for transport, nutritious food, and cloths) and physical accompaniment to ANC visit, was highly valued. Similar findings were reported in South Africa, where women indicated that their partners would bring them food while they waited for ANC services [10]. Other African research further stressed men’s role as breadwinners, responsible for securing essential items for the delivery and ensuring the availability of sufficient funds in cases of emergencies [14,16]. Furthermore, women believed that male partners benefit from attending ANC to gain knowledge about pregnancy risks (e.g., hypertension, diabetes) and to facilitate couple HIV testing, which aligns with findings in other African contexts [9,11,14,16,43]. For many, joint HIV testing at the clinic was preferred because it allowed for shared understanding and protected women from having to initiate the difficult conversation alone; furthermore, some women were afraid to tell their partner that they were HIV-positive and felt that it was better for him to hear it at the clinic.

As with similar research in South Africa [9] and Africa [11,12,13], slightly more than three quarters of the survey participants felt that it was important for men to be present in the delivery room. Logistic regression analysis found that women were more likely to support male presence during delivery if their partner had attended ANC or a previous birth, and this preference increased with maternal age. Similarly, a Nigerian study found that age, male partner presence, support at previous pregnancies, and having a male partner attend an ANC visit were significant predictors for male partner presence during the delivery [13]. Qualitative insights highlighted protection against mistreatment and, fears of baby swopping as key reasons for male presence at the delivery. Other South African research also referred to midwives’ neglectful and abusive behaviour during delivery [44,45]. Furthermore, and as reported in other African research [12], the women noted that it was important for their partners to be present at the delivery in case of an emergency (e.g., a caesarean section) where he would be required to give consent.

However, a number of women opposed male presence during labour, citing fears that men might become distressed or distract healthcare staff. A Nigerian study reported similar findings, where women indicated that men would cry during the delivery and disturb the healthcare providers [13]. Furthermore, the qualitative data also explained that men did not need to see what happened in the delivery room, as it was a woman’s secret. Research from other African countries supported this finding, where women stated that what happened in the delivery room was women’s business [11,13]. Numerous women highlighted the concern that men who saw their partner’s suffering during childbirth, would use this to verbally abuse them in future. Fears of partner violence and other negative experiences were also identified in another South African [10], with data from Sub-Saharan Africa showing a high prevalence (41.9%) of intimate partner violence among pregnant women [46].

A central insight from our study is that MPI is not universally desired—it is conditional. Many women valued involvement when it enhanced emotional security, practical assistance, and shared understanding, but they rejected more intrusive or unequal forms of engagement. Traditional gender roles continue to influence these preferences: for instance, some women believed that men should not necessarily accompany them to ANC because of potential cultural interpretations (such as “bewitching”) or to preserve women’s social spaces [6,7,14]. The desire to maintain autonomy, protect private dialogue at clinics, and avoid reinforcing patriarchal norms emerged as important considerations.

The GEM Scale data complement these qualitative insights. Participants overwhelmingly rejected violence-supporting norms. This near-universal disagreement, however, could reflect a social desirability bias given the sensitive nature of the items and the fieldworker-administered survey. In contrast, responses to items about traditional domestic roles (e.g., household duties, spousal hierarchy) were more varied. Some women continue to endorse the idea that a woman’s role includes home care, or that she should obey her husband. These mixed attitudes may underpin their nuanced views on MPI, supportive in some contexts, but not all.

The strengths of this study include the use of mixed methods to collect data, i.e., a survey and focus group discussions, as well as different participant groups, i.e., women with a child younger than six years of age as well as women of reproductive age (18–49 years), which allowed for a more in-depth understanding of women’s preferences for MPI in ANC and delivery. However, as with all research, this study has limitations. The cross-sectional design did not allow for the interpretation of causality; however, we attempted to mitigate this by triangulating the quantitative and qualitative data to gain a more in-depth understanding of women’s preferences for MPI in maternal health. In addition, there is a possibility of response bias as fieldworkers administered the survey questionnaire. As we did not specifically include pregnant women in our sample, it is recommended that future research focus more in-depth on the preferences of pregnant women for MPI in maternal health.

## 5. Conclusions

This study highlights that Sesotho-speaking women’s preferences for MPI in maternal health are supportive overall, but strongly affected by context, cultural norms, and personal comfort. Support for male presence during childbirth was more conditional and influenced by previous experience, age, and concerns about protection, privacy, and gendered expectations. The mixed endorsement of traditional gender norms further illustrates the complexity of women’s perspectives and reinforces the need for approaches that respect women’s autonomy.

Strengthening MPI initiatives therefore requires context-sensitive strategies that promote supportive and equitable engagement, rather than one-size-fits-all expectations. Programmes should encourage male attendance at ANC in ways that strengthen emotional and practical support, while respecting women’s privacy and autonomy. Health services should also address institutional barriers and cultural fears (e.g., “bewitching,” disrespect in the delivery room) by providing culturally competent counselling and flexible engagement strategies. Policies that are context-sensitive, culturally competent, and women-centred can further ensure that male involvement enhances support without compromising autonomy. Future research should focus on pregnant women specifically and examine how preferences for male involvement evolve during pregnancy and across different cultural settings.

## Figures and Tables

**Table 1:** **Participant characteristics**.

Variable	*n*	%
**Education**	N = 505	
Primary School (Grade 1–7)	16	3.2
Secondary School (Grade 8–12)	428	84.7
Tertiary education	61	12.1
**Employment status**	N = 509	
Employed/earning a salary or wage	100	19.6
Self employed	18	3.5
Unemployed and looking for work	360	70.7
Unemployed and not looking for work	26	5.1
Unable to work	5	1.0
**Social grants**	N = 499	
Receives a social grant	460	92.2
Does not receive a social grant	39	7.8
**Marital and relationship status**	N = 510	
Married	89	17.5
Not married but in a relationship	340	66.7
Single	81	15.9
**Cohabitation**	N = 423	
Living with father of youngest child	258	61.0
Not living with father of youngest child	165	39.0

**Table 2:** **Gender equitable men scale**.

Item	Agree *n* (%)	Partially Agree *n* (%)	Disagree *n* (%)
**Sub-scale: Inequitable gender norms**			
A woman who has sex before she marries does not deserve respect	23 (4.5)	2 (0.4)	488 (95.1)
It is a woman’s responsibility to avoid getting pregnant	125 (24.4)	30 (5.8)	358 (69.8)
A real man produces a male child	54 (10.5)	5 (1.0)	454 (88.5)
A woman’s role is taking care of her home and family	213 (41.5)	30 (5.8)	270 (52.6)
The husband should decide to buy the major household items	68 (13.3)	25 (4.9)	420 (81.9)
A man should have the final word about decisions in his home	55 (10.7)	13 (2.5)	445 (86.7)
A woman should obey her husband in all things	91 (17.7)	30 (5.8)	392 (76.4)
**Sub-scale: (Gender-based) Violence**			
There are times when a woman deserves to be beaten	12 (2.3)		501 (97.7)
A woman should tolerate violence to keep her family together	8 (1.6)	1 (0.2)	504 (98.2)
It is alright for a man to beat his wife/partner if she is unfaithful	9 (1.8)		504 (98.2)
A man can hit his wife/partner if she won’t have sex with him	3 (0.6)		510 (99.4)
If someone insults a man, he should defend his reputation with force if he has to	7 (1.4)	1 (0.2)	505 (98.4)
A man using violence against his wife/partner is a private matter that shouldn’t be discussed outside the couple	9 (1.8)		504 (98.2)

**Table 3:** **Women’s perspectives of MPI in ANC**.

Variable	*n*	%
**Partners accompany women to ANC** (N = 510)		
It is important for men to accompany their wives to ANC	488	95.7
It is not important for men to accompany their wives to ANC	22	4.3
**Partners encourage women to attend ANC** (N = 513)		
Partner encouraged me to go for ANC	423	82.5
Partner did not encourage me to go for ANC	90	17.5
**Partner presence at ANC visits** (N = 504)		
Partner present at ANC visits	275	54.6
Partner not present at ANC visits	229	45.4
**Partner support during pregnancy** (N = 513)		
None	64	12.5
Financial	433	84.4
Nutritious food	339	77.2
Help with household chores	285	55.6
Emotional	381	74.3
Material needs (e.g., clothes)	339	66.1

**Table 4:** **Women’s perspectives on delivery**.

Variable	*n*	%
**Men present during the delivery** (N = 508)		
It is important to for men to be present during the delivery	396	78.0
It is not important to for men to be present during the delivery	112	22.0
**Father present in the delivery room** (N = 507)		
Father was present at the delivery of his youngest child	75	14.8
Father was not present at the delivery of his youngest child	432	85.2
**Reasons for not being present in the delivery room** (N = 432)		
He did not want to be there	66	15.3
I did not want him there	10	2.3
I had to have a caesarean section	16	3.7
Lack of transport	34	7.9
He had to work	150	34.7
He was scared	64	14.8
It is not common for men in my community to do that	19	4.4
Health care workers’ negative attitudes	7	1.6

**Table 5:** **Factors associated with women’s preference for male partners to be present at delivery**.

Variables		Unadjusted Odds Ratio 95% CI	Adjusted Odds Ratio 95% CI
Living with father of youngest child	No	1	1
	Yes	1.146 (0.715–1.837)	0.898 (0.526–1.531)
Number of biological children	One	1	1
	Two	1.002 (0.601–1.672)	0.583 (0.294–1.156)
	Three	1.391 (0.757–2.555)	0.831 (0.332–2.081)
	Four+	0.954 (0.489–1.861)	0.376 (0.140–1.008)
Father attended ANC	No	1	1
	Yes	1.929 (1.256–2.963)	1.914 (1.149–3.188)
Father present at previous delivery	No	1	1
	Yes	4.529 (1.780–11.523)	4.544 (1.580–13.070)
Age		1.043 (1.011–1.077)	1.067 (1.014–1.123)
Inequitable gender norms		0.945 (0.890–1.003)	0.947 (0.883–1.015)

**Table 6:** **Themes, sub-themes and descriptors**.

Theme	Sub-Theme	Descriptor
Emotional support	Bonding during pregnancy	Early emotional connection between father and unborn baby.
	Feeling loved and supported	Women’s feelings of comfort, love and emotional reassurance during pregnancy.
	Reduced stress and mental well-being	Describes how partner involvement reduces stress, depression, and mental burden.
Practical support	Transport and hospital/clinic attendance	Describes assistance with travel and presence during clinic visits or delivery.
	Household support and shared responsibilities	Describes help with household chores and tasks.
	Physical care	Describes direct physical support such as massage, comfort, and caregiving.
	Financial support	Providing money for nutrition, health, and pregnancy needs.
Knowledge and awareness	Learning about pregnancy and baby care	Fathers attending clinics to learn how to care for mother and baby.
	Understanding health risks	Increased awareness of pregnancy-related medical complications.
	HIV testing and disclosure	Joint testing, understanding of HIV status, and shared responsibility
Protective role during delivery	Protect the newborn	Ensuring newborn safety and preventing baby swapping/theft.
	Protection from abuse and neglect	Fathers witnessing or protecting women from mistreatment in facilities.
	Legal support	Fathers signing consent forms for C-section or complications.
Conditional preferences	Privacy concerns at clinics	Sensitivity around shared stories and discomfort for men in clinic spaces.
	Discomfort with partner in delivery room	Reluctance or fear of partner presence due to judgment, embarrassment or abuse.
Cultural concerns	Community Perceptions of Male Involvement	Beliefs about witchcraft, jealousy, and stigma against supportive men.

## Data Availability

The raw data supporting the conclusions of this article will be made available by the authors upon request.
